# Development and Comparative Evaluation of Low-Cost Ultrasound-Guided Regional Anesthesia Phantom Models

**DOI:** 10.3390/gels12050388

**Published:** 2026-05-01

**Authors:** Melikşah Soylu, Mustafa Azizoğlu

**Affiliations:** 1 Department of Anaesthesiology and Intensive Care, Aksaray Education and Research Hospital, Aksaray 68200, Turkey; meliksahsoylu@gmail.com; 2 Department of Anaesthesiology and Intensive Care, Faculty of Medicine, Mersin University, Mersin 33343, Turkey

**Keywords:** regional anesthesia, ultrasound-guided regional anesthesia, phantom models, medical education, simulation

## Abstract

Regional anesthesia is vital for modern surgical practices, but accessibility to training is often hampered by the high cost of commercial phantom models. This study aimed to develop and evaluate low-cost, realistic phantom alternatives using Ecoflex, borax-containing polyvinyl alcohol (PVA), and plastisol compositions. The models were evaluated under ultrasound for imaging properties, including needle visibility, tissue resistance, cost, contrast-to-noise ratio (CNR), signal-to-noise ratio (SNR), axial full width at half maximum (FWHM), and compared to a commercial reference (Blue Phantom). Initial qualitative assessments were performed by three experienced evaluators, and inter-observer agreement demonstrated good to excellent reliability. In addition, a long-term usability assessment was conducted more than one year after phantom preparation, involving 20 participants using a structured Likert scale. A statistically significant difference was observed among materials (Friedman test, *p* < 0.05), with PVA hydrogel containing 20 g borax and the Blue Phantom demonstrating the highest tissue realism scores, without a significant difference between them. The results showed that plastisol softener and PVA (20 g borax) hydrogel provided excellent needle visibility and tissue resistance and achieved an imaging performance comparable to the commercial model. Notably, CNR and SNR values for these materials approached reference levels, while costs ranged from $0.5 to $2.50 per 100 mL, representing a significant reduction compared to $45 per 100 mL for commercial models. In conclusion, this research confirms that affordable materials such as PVA and plastisol can effectively simulate human tissue for ultrasound-guided training. Furthermore, the findings suggest that PVA-based hydrogels may provide sustained usability over time, offering a practical and accessible solution for enhancing clinical skill acquisition in resource-constrained settings.

## 1. Introduction

Regional anesthesia provides patients with a pain-free surgical experience [1], enhances comfort, reduces anesthesia-related risks, and facilitates postoperative recovery [2]. The success of these techniques depends largely on the clinician’s ability to accurately identify anatomical structures and precisely deliver local anesthetic agents to the intended target site [3,4]. The introduction of ultrasound guidance has represented a major advancement in regional anesthesia, reducing block performance time, local anesthetic volume, and complication rates compared with traditional landmark-based techniques [5].

As ultrasound-guided regional anesthesia (UGRA) becomes standard practice, structured and reproducible training is essential. In recent years, the growing emphasis on patient safety and comfort has accelerated the integration of simulation-based education into medical training, increasingly supplementing traditional apprenticeship models. Among the tools used in simulation-based learning, medical phantoms—physical models designed to replicate human anatomical and physiological characteristics—play a critical role in procedural skill acquisition [6].

Phantom-based simulation offers a controlled and risk-free environment in which trainees can develop psychomotor skills, hand–eye coordination, and technical proficiency prior to clinical application [7,8]. In ultrasound-guided regional anesthesia, the ability to visualize the needle and interpret sonographic anatomy is a critical technical skill for anesthesiologists. Simulation models therefore play an important role in early training by allowing for repeated practice of needle–probe coordination before performing procedures on patients. Developing accessible phantom models may contribute to safer skill acquisition and broader implementation of ultrasound-guided regional anesthesia training. Professional societies, including the American Society of Regional Anesthesia and Pain Medicine (ASRA) and the European Society of Regional Anaesthesia and Pain Therapy (ESRA), emphasize the importance of simulation-based education in achieving competency in UGRA [9].

An ideal phantom should closely mimic human tissue properties, be affordable and easily accessible, allow for repeated use, provide realistic tactile feedback, and pose no health risk to the user [10]. For peripheral nerve block training, additional requirements include clear ultrasound visualization of the needle, tissue resistance comparable to in vivo conditions, adequate needle stability, and minimal persistent needle track formation.

Despite these educational needs, current training frequently relies either on direct patient experience or on commercially manufactured phantom models, which may be associated with substantial financial costs and limited accessibility, particularly in resource-constrained settings [11,12]. Commercial ultrasound training phantoms are marketed within a broad price range, from approximately 620 USD to over 50,000 USD [13].

The aim of this study was to systematically compare multiple low-cost phantom materials under standardized ultrasound conditions, including the evaluation of different borax concentrations in PVA-based hydrogels. By integrating objective ultrasound imaging metrics, including contrast-to-noise ratio (CNR), signal-to-noise ratio (SNR), and axial full width at half maximum (FWHM), with user-based assessments such as needle visibility and tactile realism, this study aims to identify a practical and optimized phantom model for ultrasound-guided regional anesthesia training. To our knowledge, few studies have performed a structured and quantitative comparison of low-cost phantom materials using both imaging-based metrics and user-centered evaluations. Therefore, this study also seeks to provide a practical framework for the evaluation and selection of low-cost ultrasound phantoms in training settings. We hypothesized that selected low-cost materials could provide sonographic visibility and needle-tissue interaction characteristics comparable to commercially available models, while significantly reducing overall cost.

## 2. Results and Discussion

**Figure 1:**
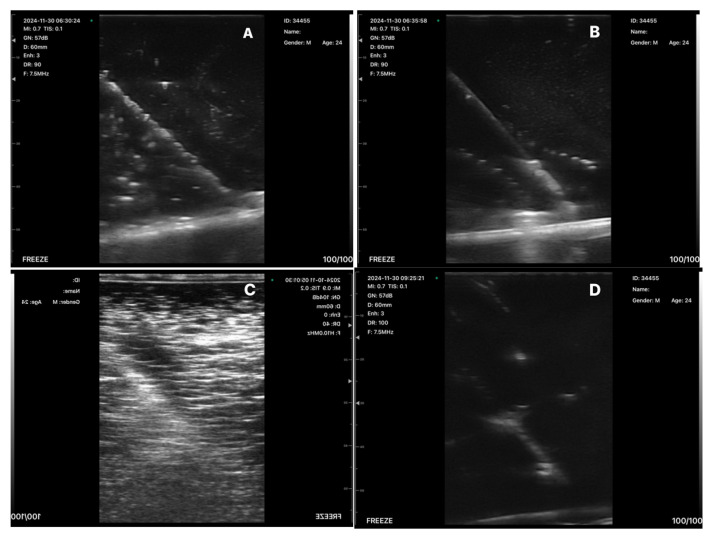

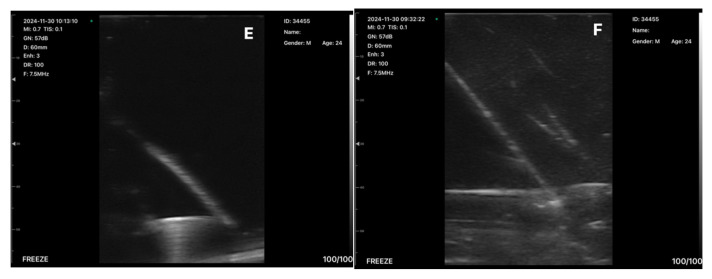
Ultrasound images showing the needle and wire together at a depth of 5 cm. (**A**): PVA + 20 g borax mixture; (**B**): PVA + 5 g borax mixture; (**C**): Ecoflex gel; (**D**): plastisol; (**E**): plastisol + softener; (**F**): Blue Phantom.

In the material made with Ecoflex gel, needle visualization at 5 cm depth was technically challenging, and no persistent needle tract was observed after advancement (Figure 1C). Although the material demonstrated good mechanical durability over one month, ultrasound image quality was limited.

In PVA-based phantoms, both formulations allowed for visualization of the needle and embedded wire at 5 cm depth (Figure 1A). The PVA + 20 g borax mixture demonstrated realistic tactile resistance during needle advancement, closely resembling human soft tissue. A temporary needle tract remained after insertion and disappeared within approximately 4 h, indicating self-healing properties. Prolonged probe pressure caused transient deformation; however, the material recovered its original shape within approximately 10 min. Samples stored at room temperature with the lid closed maintained structural integrity for at least one month. In contrast, the PVA + 5 g borax formulation exhibited reduced mechanical stability with rapid deformation under probe pressure and lower perceived tissue resistance (Figure 1B).

Plastisol-based phantoms provided good needle visualization at 5 cm depth (Figure 1D). Pure plastisol exhibited firmer tactile resistance compared with human soft tissue and demonstrated persistent needle tract formation after needle withdrawal. The plastisol + softener formulation improved perceived tissue realism and reduced needle tract formation; however, axial image quality was inferior compared with pure plastisol (Figure 1E).

The commercial Blue Phantom allowed for easy needle visualization at 5 cm depth with persistent needle tract formation and excellent long-term durability (Figure 1F).

Tissue realism and tactile resistance were evaluated using a structured Likert-based scoring system, as summarized in Table 1. Each material was assessed independently by three experienced operators, with three repeated evaluations per material. The reported values represent the median scores of these repeated assessments. Inter-rater reliability demonstrated good to excellent agreement, with an average-measures ICC of 0.88 (95% CI: 0.53–0.98) supporting the consistency and reproducibility of the subjective assessments.

Cost analysis standardized per 100 mL revealed substantial differences between materials. PVA-based formulations were the most economical (approximately 0.5 USD per 100 mL), followed by plastisol and plastisol with softener (approximately 2.5 USD per 100 mL) and Ecoflex (approximately 7 USD per 100 mL). The commercial Blue Phantom represented the highest cost option (approximately 40 USD per 100 mL) (Table 1).

This table summarizes the ultrasound visibility performance, subjective tactile realism (Likert scale, 1–5), and cost efficiency of each phantom material. Values represent the median (IQR) from expert-based assessment.

Final usability assessment was conducted more than one year after phantom preparation, independently from the initial expert-based evaluation phase. Likert-scale evaluations obtained from 20 participants are presented as the median (interquartile range) in Table 2.

A statistically significant difference was observed among the materials (Friedman test, χ^2^ = 80.65, *p* < 0.05). The highest tissue realism scores were observed for PVA + 20 g borax [4 (4–5)] and the Blue Phantom [4 (4–5)], with no statistically significant difference between these two materials. Materials marked with an asterisk (*) in Table 2 demonstrated significantly lower tissue realism scores compared to the Blue Phantom (*p* < 0.05, Bonferroni-corrected Wilcoxon signed-rank test). Specifically, plastisol + softener [3 (3–4)], plastisol [3 (2–3)], Ecoflex [3 (2–3)], and PVA + 5 g borax [2 (2–3)] showed significantly lower scores relative to the reference model (see Supplementary Table S1).

Quantitative ultrasound image quality metrics (CNR, SNR, and FWHM) and subjective assessments of needle visualization and tissue resistance after one year are presented in Table 2. The Blue Phantom demonstrated the highest signal-to-noise ratio (SNR: 6.87 ± 1.60) and contrast-to-noise ratio (CNR: 3.03 ± 0.49), serving as the reference standard. Among the experimental materials, PVA + 20 g borax achieved axial spatial resolution comparable to the Blue Phantom (0.30 ± 0.02 mm vs. 0.32 ± 0.06 mm) and exhibited the highest CNR (1.56 ± 0.18) and SNR (2.55 ± 0.10) among low-cost phantoms. PVA + 5 g borax showed the poorest imaging performance (SNR: 1.17 ± 0.06; CNR: 0.11 ± 0.02; FWHM: 1.73 ± 0.15 mm). Ecoflex demonstrated moderate SNR (4.88 ± 0.46) but inferior contrast and axial resolution (CNR: 0.38 ± 0.01; FWHM: 1.52 ± 0.22 mm). Plastisol exhibited intermediate imaging performance (SNR: 1.63 ± 0.85; CNR: 0.56 ± 0.09; FWHM: 0.63 ± 0.06 mm), while the addition of softener further degraded axial resolution (0.93 ± 0.04 mm) despite slightly improved SNR: 1.83 ± 0.96 and CNR: 0.60 ± 0.10.

This study compared multiple low-cost phantom materials in terms of ultrasound image quality, tactile realism, durability, and cost efficiency at a standardized needle depth of 5 cm. The main finding was that PVA combined with 20 g borax provided the most balanced performance, demonstrating axial spatial resolution comparable to the commercial Blue Phantom while maintaining realistic tissue resistance and extremely low production cost. As shown in Table 2, this material exhibited higher relative CNR and SNR values and lower FWHM compared to other low-cost materials, while maintaining realistic tissue resistance and extremely low production cost. These findings were consistent across repeated measurements. Increasing borax concentration resulted in a more stable product, which improved structural integrity and enhanced the similarity of tactile feedback to human soft tissue. Although the commercial Blue Phantom achieved the highest SNR and CNR values, this was expected, as commercially manufactured phantoms are optimized for ultrasonographic consistency and durability. However, their substantially higher cost limits widespread accessibility, particularly in resource-constrained educational settings.

Specifically, the PVA + 20 g borax formulation provided superior stability and more realistic needle resistance compared to the 5 g formulation, while maintaining adequate ultrasound visibility. Plastisol alone was found to be suboptimal in terms of tissue realism. However, when combined with a softener, the resulting material more closely approximated the tactile characteristics of human tissue while preserving satisfactory ultrasound needle visualization. This suggests that mechanical property modulation through plasticizer addition is critical for optimizing phantom realism. In contrast, silicone-based materials demonstrated limitations. Although lower Shore hardness silicone formulations partially resembled human tissue in terms of resistance, ultrasound image quality was insufficient due to artifact formation and poor needle conspicuity. Therefore, despite acceptable mechanical durability, silicone-based phantoms were less suitable for ultrasound-guided needle training in their tested configurations. PVA-based hydrogels have previously been described as effective tissue-mimicking materials for ultrasound-guided needle training because of their tunable stiffness, favorable imaging properties, and self-healing behavior [14,15]. Increasing borax concentration enhances cross-link density, improving mechanical stability and reducing excessive deformation under probe pressure [16], which likely explains the superior performance of the 20 g formulation compared with the 5 g mixture. In our study, this effect was reflected in the improved imaging performance of the PVA + 20 g borax formulation compared to the 5 g formulation, with higher CNR and SNR values and improved axial resolution.

Plastisol provided acceptable needle visualization and moderate axial resolution; however, tactile resistance was perceived as firmer than human soft tissue. Polyvinyl chloride plastisol (PVCP)-based phantoms are widely reported as durable and reproducible materials with adjustable mechanical properties via plasticizer modification [17,18,19]. Nevertheless, their imaging performance may not fully replicate soft tissue characteristics, particularly when stiffness is increased. The plastisol + softener formulation improved subjective tissue realism but demonstrated degraded axial resolution compared with pure plastisol. This suggests that mechanical tuning may negatively influence ultrasonographic beam characteristics, highlighting the inherent trade-off between tactile realism and image sharpness for regional anesthesia.

Ecoflex silicone demonstrated poor contrast resolution and inferior axial spatial resolution. Silicone-based materials are known for mechanical durability; however, their intrinsic scattering properties may limit needle conspicuity unless specifically engineered for ultrasound applications [20].

Anesthesiologists must acquire the technical and practical skills required for regional anesthesia with precision and competence [21]. In this context, establishing a safe and appropriate training environment is of paramount importance. Traditional patient-based training methods may compromise patient safety and comfort, particularly during the early stages of skill acquisition [22]. Phantom models, therefore, provide residents with the opportunity to consolidate these skills in a risk-free environment before performing procedures on patients.

Moreover, the widespread implementation of phantom-based simulation may facilitate the standardization of training processes across institutions [23,24]. In addition, simulation training contributes to psychological preparedness, allowing trainees to build confidence before transitioning to real clinical settings [25].

In the present study, phantom models produced from five different materials were evaluated under ultrasound guidance. Two PVA-based mixtures containing 5 g and 20 g borax were comparatively assessed. The 20 g formulation demonstrated superior stability and improved image quality [26,27]. Among all tested materials, PVA + 20 g borax appeared to be the most favorable phantom model in the present experimental setting.

Adequate needle visualization is essential for effective training, as it allows practitioners to refine hand–eye coordination and needle control in a controlled environment that closely mimics clinical practice. The self-healing property of PVA hydrogel permits needle tracks to disappear over time, thereby enhancing durability and repeated usability, making it particularly suitable for iterative training sessions [28].

Importantly, with a cost of less than 1 USD per 100 mL, the PVA hydrogel-based phantom may represent a highly accessible solution, especially for resource-limited and developing settings. Its low cost, ease of preparation, and acceptable realism make it a practical and reassuring training tool for novice practitioners.

Minton et al. increased the concentration of PVA and applied repeated freeze–thaw cycles to enhance the stiffness and mechanical strength of the phantom material. In contrast, in the present study, stiffness modulation was achieved by altering borax concentration.

Compared to freeze–thaw-based phantom production, the addition of borax considerably shortened and simplified the preparation process. The freeze–thaw technique requires controlled temperature cycling and prolonged preparation time, whereas borax-induced crosslinking allows for rapid gel formation under room conditions. This practical advantage may be particularly relevant for institutions seeking easily reproducible and time-efficient phantom production.

Furthermore, the more heterogeneous internal structure observed after borax addition may better approximate human soft tissue, which is inherently heterogeneous in composition and mechanical behavior [29]. This structural variability may contribute to a more realistic tactile experience during needle advancement.

In this study, no additional foreign scattering agents were incorporated to enhance ultrasound image quality. However, to further optimize imaging backscatter properties, materials such as talcum powder or graphite may be added to the PVA matrix in future applications. Such additives could improve echogenicity and potentially refine needle conspicuity under ultrasound guidance without substantially increasing cost.

The plastisol + softener mixture emerged as an effective model in terms of both tissue resistance and ultrasound image quality. This formulation not only allowed for clear needle visualization but also minimized persistent needle tract formation. Reduced needle track visibility represents an advantage for repeated use. However, unlike PVA-based models, plastisol does not exhibit true self-healing properties; therefore, needle tracks may accumulate over time, potentially reducing long-term performance. In addition to these differences, needle insertion-related material behavior also varied between phantom types. In PVA-based phantoms, needle advancement resulted in temporary tract formation that gradually disappeared over time, indicating a self-healing property likely related to the hydrogel’s viscoelastic and water-retaining structure. In contrast, plastisol-based materials exhibited persistent needle tracts following needle withdrawal, suggesting cumulative structural alteration with repeated use.

These observations are particularly relevant for training applications, as repeated needle insertions are inherent to simulation-based learning. Materials with self-healing properties may better support repeated practice sessions without progressive degradation of structural integrity or image quality. However, it should be emphasized that these findings are based on qualitative observations rather than a predefined durability testing protocol.

The similarity of needle resistance to human tissue is particularly important for trainees, as realistic tactile feedback facilitates the acquisition of fine motor skills and enhances confidence before transitioning to real patients [30].

In contrast, the PVA + 5 g borax mixture failed to provide a sufficiently stable surface due to its softer consistency. Under ultrasound probe pressure, tissue deformation occurred readily, which may negatively affect training quality—especially during precision-dependent procedures. Mechanical instability can impair accurate needle control practice and may not adequately simulate realistic tissue behavior.

Pepley et al. produced phantoms by incorporating softeners into PVC and reported that the softened model performed better than rigid formulations [31]. They also compared their PVC-based phantom with a commercial model and found comparable performance [31]. These findings are consistent with the present study, in which plastisol combined with a softener improved both ultrasound image quality and tactile realism.

Qurash et al. developed a gelatin-based phantom and compared it with a commercial Blue Phantom in terms of cost. Similarly, Sultan et al. reported that gelatin-based phantoms were reusable and durable [24]. Although gelatin is inexpensive, its food-based composition poses potential infection risks and requires repeated heating during preparation [32]. Additionally, to prevent microbial contamination, storage under refrigeration is necessary.

In contrast, the PVA + borax material developed in our study maintains its water content at room temperature when stored in a closed container. Occasional rehydration with small amounts of water is sufficient to prolong usability [32]. Its cost-effectiveness is comparable to gelatin-based models while avoiding food-related contamination risks. Silicone- and plastisol-based materials, on the other hand, demonstrate high durability and structural stability without specific storage requirements. In addition, follow-up observations performed approximately one year after phantom preparation revealed no visible deterioration in ultrasound image quality or physical structure when the materials were stored in closed containers. While these findings suggest that such storage conditions may support medium-term stability, this observation was not based on a predefined durability testing protocol and should therefore be interpreted cautiously. When exposed to air, the materials exhibited dehydration and shrinkage; however, rehydration with water largely restored their original structure. This reversible behavior suggests that moisture content plays a key role in maintaining phantom integrity.

One of the major strengths of this study is the development of phantom models using low-cost and readily available materials. These models provide a safe simulation environment for medical students and anesthesiologists before clinical application, thereby potentially enhancing patient safety and procedural success. The affordability and reproducibility of these materials represent a substantial advantage, particularly for institutions with limited resources and for training programs in developing countries [33].

The findings of this study may provide practical guidance for researchers and educators in selecting and developing low-cost ultrasound phantom materials. The proposed evaluation approach, combining quantitative imaging metrics with user-based assessments, may serve as a reproducible framework for future studies. In addition, the identification of PVA-based hydrogels with optimized borax concentration as a cost-effective alternative to commercial models may facilitate wider access to ultrasound training tools, particularly in resource-limited settings. Final reassessment demonstrated that the materials maintained both practical usability and imaging performance under routine conditions. In this context, commonly used low-cost training models such as tofu, food-grade gelatin, and porcine tissue are known to be prone to rapid degradation, contamination, and limited shelf life, often necessitating frequent replacement and specific storage conditions [34,35]. In contrast, the PVA-based hydrogels evaluated in the present study demonstrated sustained structural integrity and stable imaging characteristics over an extended period, suggesting a more durable and practical option for repeated use in training environments. While this observation should be interpreted as application-oriented rather than a formal durability analysis, it highlights a potential advantage of these materials in terms of longevity and usability. These findings support the role of optimized PVA-based phantoms as accessible, reusable, and clinically relevant tools for ultrasound-guided procedural training.

Despite its contributions, this study has several limitations. First, although phantom models allow for standardized comparisons, they cannot fully replicate the complex ultrasonographic and mechanical properties of human tissues. In particular, quantitative mechanical parameters such as elastic modulus and viscosity were not measured, and acoustic properties such as attenuation coefficient and speed of sound were not directly evaluated. Although imaging-based metrics such as signal-to-noise ratio (SNR), contrast-to-noise ratio (CNR), and axial resolution (FWHM) provide indirect assessment of ultrasonographic performance, a full physicochemical characterization was beyond the scope of this study.

Second, the evaluation was conducted under controlled phantom conditions using a single ultrasound system; therefore, the findings may vary with different ultrasound devices, probe configurations, and real clinical environments where tissue heterogeneity and patient-related factors are present.

Third, the evaluation involved a limited number of users. Although repeated measurements and inter-observer reliability analysis were performed, future studies including a larger and more diverse group of participants are needed to better assess inter-user variability and generalizability.

Finally, certain material-specific limitations were identified. The Ecoflex gel phantom demonstrated poor needle visualization under ultrasound, limiting its suitability for training purposes. Similarly, the PVA + 5 g borax formulation exhibited insufficient structural stability, with excessive deformation under probe pressure, which may compromise training realism and procedural accuracy.

Although qualitative observations regarding needle track formation and self-healing behavior were reported, durability under repeated needle insertions was not systematically evaluated using a standardized protocol (e.g., defined insertion cycles or longitudinal performance measurements).

## 3. Conclusions

In this study, six low-cost phantom materials were evaluated under standardized ultrasound conditions. The PVA hydrogel with 20 g borax demonstrated the most favorable overall performance, with higher CNR and SNR values and lower FWHM compared to other materials, approaching the performance of the commercial reference model.

The plastisol + softener model also showed good imaging quality and realistic tactile properties, representing a viable alternative.

These findings highlight that low-cost materials can provide effective training models and offer a practical framework for the evaluation and selection of ultrasound phantom materials, particularly in resource-limited settings.

## 4. Materials and Methods

### 4.1. Material Selection

**Figure 2:**
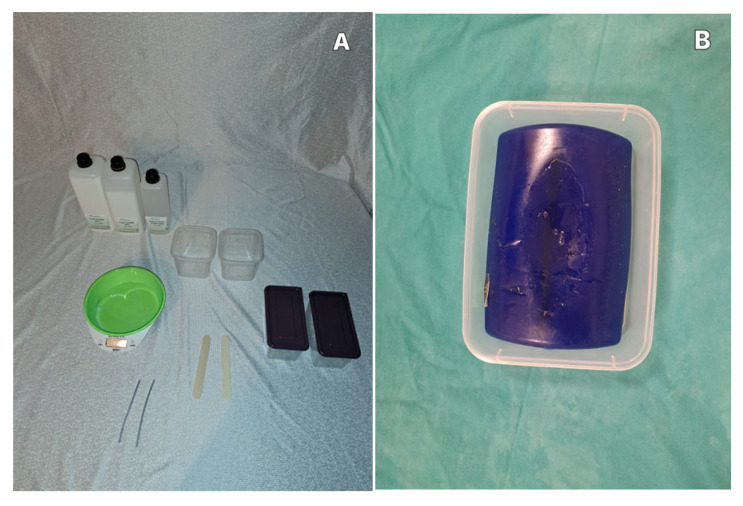
(**A**): Material preparation stages and (**B**): the finished Blue Phantom.

A comprehensive literature review and online content search (including academic databases and publicly accessible educational platforms) were conducted by MS and MA to identify materials previously used in the construction of low-cost ultrasound-compatible phantom models. Based on this review, plastisol, platinum-cured silicone (Ecoflex), and polyvinyl alcohol (PVA) hydrogel combined with borax were selected for experimental evaluation (Figure 2A). A commercially manufactured phantom model (Blue Phantom^®^(GTSimulators by Global Technologies, Davie, FL, USA)) was used as a reference comparator (Figure 2B). Each material was prepared in standardized plastic containers measuring 13 × 8 × 7 cm, and the preparation stages were documented photographically.

### 4.2. Phantom Preparation

The first material evaluated was platinum-cured silicone (Ecoflex^™^ 00-30; Smooth-On Inc., Macungie, PA, USA) and Ecoflex Gel™; Smooth-On Inc., Macungie, PA, USA). Both materials were supplied as two-component systems (Part A and Part B). Ecoflex gel constituted the primary bulk material; however, due to its extremely low Shore hardness (000-35, below the Shore 00 scale) and inherent surface tackiness, encapsulation with a higher Shore hardness silicone was required.

Ecoflex 00-30 (Shore 00-30) was therefore used to create structural layers. Equal parts (1:1 ratio) of Ecoflex 00-30 components were manually mixed for 30 s using a wooden spatula and immediately poured from a single point into a 13 × 8 × 7 cm container to minimize air entrapment. The material was allowed to cure for 30 min. After curing the base layer, a cable and a water-filled rubber tube were positioned within the mold to simulate linear and tubular anatomical structures. Subsequently, Ecoflex gel was prepared in a 1:1 ratio and poured into the container, leaving approximately one finger-width space from the upper edge. A final top layer of Ecoflex 00-30 was then prepared and applied to form a superficial covering. This outer layer was intended to reduce surface stickiness, mimic skin consistency, and provide probe stability during ultrasound examination.

Polyvinyl alcohol gel (Kimyacınız Inc., Istanbul, Türkiye) containing approximately 10% PVA was used as another phantom material. Two different borax concentrations (5 g and 20 g) were tested. For each preparation, 500 g of PVA hydrogel was transferred into a container. Borax (5 g or 20 g) was dissolved in 200 mL of water under continuous stirring until fully solubilized. The borax solution was gradually added to the PVA hydrogel while continuously mixing until the desired consistency was achieved. The mixture was then left undisturbed for one week to allow entrapped air bubbles to rise and dissipate. After this resting period, a copper wire was positioned at a depth of 5 cm to simulate an echogenic linear structure. The mixture was transferred into a standardized container. A thin superficial layer (a few millimeters) of Ecoflex 00-30 silicone was applied to the surface to create a skin-like layer, prevent excessive PVA flow, and provide a stable interface for ultrasound probe contact. Identical procedures were followed for both borax concentrations.

Plastisol (Plastileurre^®^, Bricoleurre Inc., Mont Saint Aignan, France) was prepared in two configurations: pure plastisol and plastisol mixed with softener (Assouplissant^®^, Bricoleurre Inc., Mont Saint Aignan, France) (1:1 by volume). Approximately 600 g of liquid plastisol was placed in a container and heated in a microwave oven at 600 W for 8 min. During heating, the material was removed every 2 min and stirred for 5 s. In the final 2 min, stirring was performed every 30 s for 5 s. Heating continued until the material became fully transparent. The liquefied plastisol was then poured slowly from a single point into a mold to minimize air bubble formation and left to cool for 2 h until solidified. For the plastisol–softener mixture, the same heating and pouring procedures were followed. In both plastisol models, a copper wire was positioned at a depth of 5 cm prior to final solidification.

The prepared materials were stored in sealed containers of defined dimensions under room conditions. No visible structural or imaging-related deterioration was observed over several months. In cases where partial drying occurred due to prolonged exposure to air, the materials were able to regain their original form after light rehydration.

A commercially manufactured phantom model (Blue Phantom^®^ Elevate Healthcare Inc., Sarasota, FL, USA) available in our department was used as the reference comparator.

### 4.3. Ultrasound Evaluation Protocol

**Figure 3:**
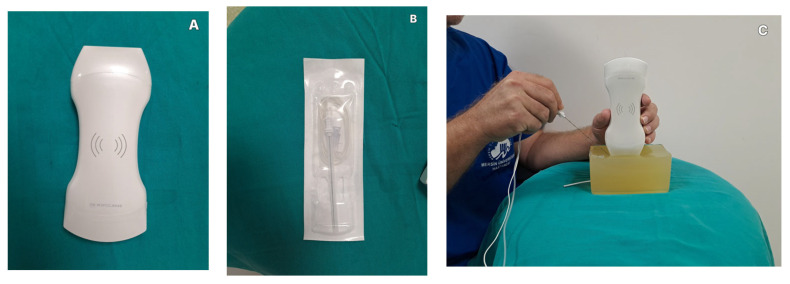
Ultrasound evaluation protocol: (**A**): Alexus A10 HRL ultrasound linear probe; (**B**): Sonoplex^®^ Stim Cannula 21-gauge, 100 mm; (**C**): needle and wire shown together at a depth of 5 cm. The probe was positioned perpendicular to the phantom surface and minimal probe pressure was applied during image acquisition. The same ultrasound preset and imaging settings were used for all acquisitions.

All phantom models were individually evaluated under ultrasound guidance. Needle visualization was assessed at a standardized depth of 5 cm, where both the needle shaft and the embedded copper wire were visualized simultaneously under ultrasound guidance to allow for consistent comparison between phantom materials. A 21-gauge, 100 mm Sonoplex^®^ Stim Cannula block needle (Pajunk Medical Products, Geisingen, Germany) (Figure 3B) was used in all evaluations. Ultrasound imaging was performed using an Alexus A10 HRL handheld ultrasound system (Alexus Inc., Istanbul, Türkiye) (Figure 3A). A linear probe was used with a frequency of 10–12 MHz. The imaging depth was set to 7 cm, and the gain was adjusted to approximately 60 dB. The dynamic range was set to 90 dB. Needle visualization was assessed at insertion. Visualization of a copper wire positioned at approximately 5 cm depth was evaluated concurrently with needle advancement. (Figure 3C)

Tissue resistance encountered during needle advancement was qualitatively compared with that experienced in human tissue and scored using a 5-point Likert scale (5 = most similar; 1 = least similar). Three anesthesiologists experienced in ultrasound-guided regional anesthesia independently performed the assessments while blinded to the material type. All measurements were performed three times in succession, and the reported values represent the median of these repeated assessments. Inter-observer reliability was evaluated using the intraclass correlation coefficient (ICC).

To assess final usability and imaging stability, a secondary evaluation was conducted more than one year after phantom preparation. Ultrasound images were re-acquired and quantitatively analyzed, and user-based evaluations were performed by 20 participants using a structured 5-point Likert scale under the same standardized conditions. Ultrasound imaging was performed using a Mindray Consona N8 ultrasound system (Shenzhen, China). A linear probe was used with a frequency of 3–13 MHz. All images were acquired using the same imaging parameters to ensure consistency across measurements. Real-time images were transmitted to a tablet device and digitally recorded. All image measurements were performed by a single investigator to ensure measurement consistency.

Image quality was evaluated using contrast-to-noise ratio (CNR), signal-to-noise ratio (SNR), and axial resolution measurements on each phantom image. Three B-mode images were acquired under identical system settings. Image analysis was performed using ImageJ software (Version 1.53t, National Institutes of Health, Bethesda, MD, USA). Regions of interest (ROIs) measuring 30 × 30 pixels were manually positioned on the needle tip and on a homogeneous background area at the same depth. Mean gray-level intensity and standard deviation values were extracted for subsequent CNR and SNR calculations.

CNR was calculated using the combined standard deviation method:

$$CNR=\frac{|\mu_{target}-\mu_{background}|}{\sqrt{\sigma_{target}^{2}+\sigma_{background}^{2}}}$$

where *μ* represents mean gray-level intensity and *σ* denotes standard deviation.

Background SNR was calculated as:

$$SNR=\frac{\mu_{background}}{\sigma_{background}}$$

Axial resolution was quantified by calculating the full width at half maximum (FWHM) of the axial intensity profile along the ultrasound beam direction at the needle location. The intensity profile was obtained using a line profile drawn along the depth axis through the center of the needle echo. Measurements were initially obtained in pixel units and subsequently converted to millimeters using the spatial scale provided by the ultrasound image. Quantitative ultrasound measurements (CNR, SNR, and axial resolution) were performed after a dedicated methodological development and standardization phase, during which appropriate image analysis techniques were identified and implemented. Following this preparatory period, final measurements were conducted approximately one year after phantom preparation to ensure consistency and reproducibility of the analysis under stabilized conditions.

### 4.4. Statistical Analysis

Statistical analyses were performed using MedCalc Statistical Software (MedCalc Software Ltd., Ostend, Belgium). Qualitative assessments of tissue resistance were performed independently by three blinded evaluators using a 5-point Likert scale, and the results are reported as median and interquartile range. Inter-observer agreement was assessed using the intraclass correlation coefficient (ICC; two-way random-effects model; absolute agreement). Image-based measurements (CNR, SNR, and axial resolution) were obtained from three repeated measurements performed by a single operator for each material, and results are presented as mean ± standard deviation. Differences among materials were assessed using the Friedman test. When a statistically significant difference was detected, post hoc pairwise comparisons were performed using the Wilcoxon signed-rank test with Bonferroni correction. A *p*-value < 0.05 was considered statistically significant. Descriptive statistics were used to summarize the results.

## Figures and Tables

**Table 1:** General characteristics of the tested phantom materials with initial expert-based evaluation.

Material	Needle Visibility	Tissue Realism (1–5) n = 3	Cost (USD/100 mL)	Cost Ratio vs. Blue
Ecoflex	Poor	3 (2–4)	7	5.7× cheaper
PVA hydrogel +20 g Borax	Good	4 (3–5)	0.5	80× cheaper
PVA hydrogel +5 g Borax	Good	2 (1–3)	0.5	80× cheaper
Plastisol	Moderate	3 (2–4)	2.5	16× cheaper
Plastisol + Softener	Good	4 (3–5)	2.5	16× cheaper
Blue Phantom	Good	3 (2–5)	40	Reference

**Table 2:** Ultrasound metrics and final comparison results of phantom materials.

Material	CNR	CNR (% of Blue)	SNR	SNR (% of Blue)	FWHM (mm)	Final Tissue Realism Evaluation (1–5) n = 20
Ecoflex	0.38 ± 0.01	13%	4.88 ± 0.46	71%	1.52 ± 0.22	3 (2–3) *
PVA hydrogel +20 g Borax	1.56 ± 0.18	51%	2.55 ± 0.10	37%	0.30 ± 0.02	4 (4–5)
PVA hydrogel +5 g Borax	0.11 ± 0.02	4%	1.17 ± 0.06	17%	1.73 ± 0.15	2 (2–3) *
Plastisol	0.56 ± 0.09	18%	1.63 ± 0.85	24%	0.63 ± 0.06	3 (2–3) *
Plastisol + Softener	0.60 ± 0.10	20%	1.83 ± 0.96	27%	0.93 ± 0.04	3 (3–4) *
Blue Phantom	3.03 ± 0.49	100%	6.87 ± 1.60	100%	0.32 ± 0.06	4 (4–5)

Data are presented as median (interquartile range). Group comparisons were performed using the Friedman test (χ^2^ = 80.65, *p* < 0.05), followed by Bonferroni-corrected Wilcoxon signed-rank tests for pairwise comparisons. * indicates a statistically significant difference compared to the reference group (Blue Phantom) (*p* < 0.05).

## Data Availability

The original contributions presented in this study are included in the article/Supplementary Material. Further inquiries can be directed to the corresponding author.

## Supplementary Materials

The following supporting information can be downloaded at: https://www.mdpi.com/article/10.3390/gels12050388/s1. Table S1. Pairwise comparisons between materials; Figure S1. Long-term appearance and ultrasound characteristics of phantom materials.

## Abbreviations

USG	Ultrasonography
PVA	Polyvinyl alcohol
