# Supplementary material for: Development and Comparative Evaluation of Low-Cost Ultrasound-Guided Regional Anesthesia Phantom Models
Source: Gels. 2026 May 1;12(5):388. doi: 10.3390/gels12050388 (PMC13206265; doi:10.3390/gels12050388)
Supplement: Supplementary file 1 [file gels-12-00388-s001.zip › gels-4225613-supplementary.pdf]

Table S1. Pairwise comparisons between materials.

Comparison	Bonferroni-adjusted p-value	Significant at $p < 0.05$
Ecoflex vs PVA + 20 g Borax	0.00088	Yes
Ecoflex vs PVA + 5 g Borax	0.522	No
Ecoflex vs Plastisol	1.000	No
Ecoflex vs Plastisol + Softener	0.096	No
Ecoflex vs Blue Phantom	0.00155	Yes
PVA + 20 g Borax vs PVA + 5 g Borax	0.00065	Yes
PVA + 20 g Borax vs Plastisol	0.00098	Yes
PVA + 20 g Borax vs Plastisol + Softener	0.0138	Yes
PVA + 20 g Borax vs Blue Phantom	1.000	No
PVA + 5 g Borax vs Plastisol	1.000	No
PVA + 5 g Borax vs Plastisol + Softener	0.00194	Yes
PVA + 5 g Borax vs Blue Phantom	0.00103	Yes
Plastisol vs Plastisol + Softener	0.00336	Yes
Plastisol vs Blue Phantom	0.00098	Yes
Plastisol + Softener vs Blue Phantom	0.00504	Yes

Pairwise comparisons were performed using Wilcoxon signed-rank tests with Bonferroni correction. Statistical significance was $p < 0.05$.

Figure S1. Long-term appearance and ultrasound characteristics of phantom materials.

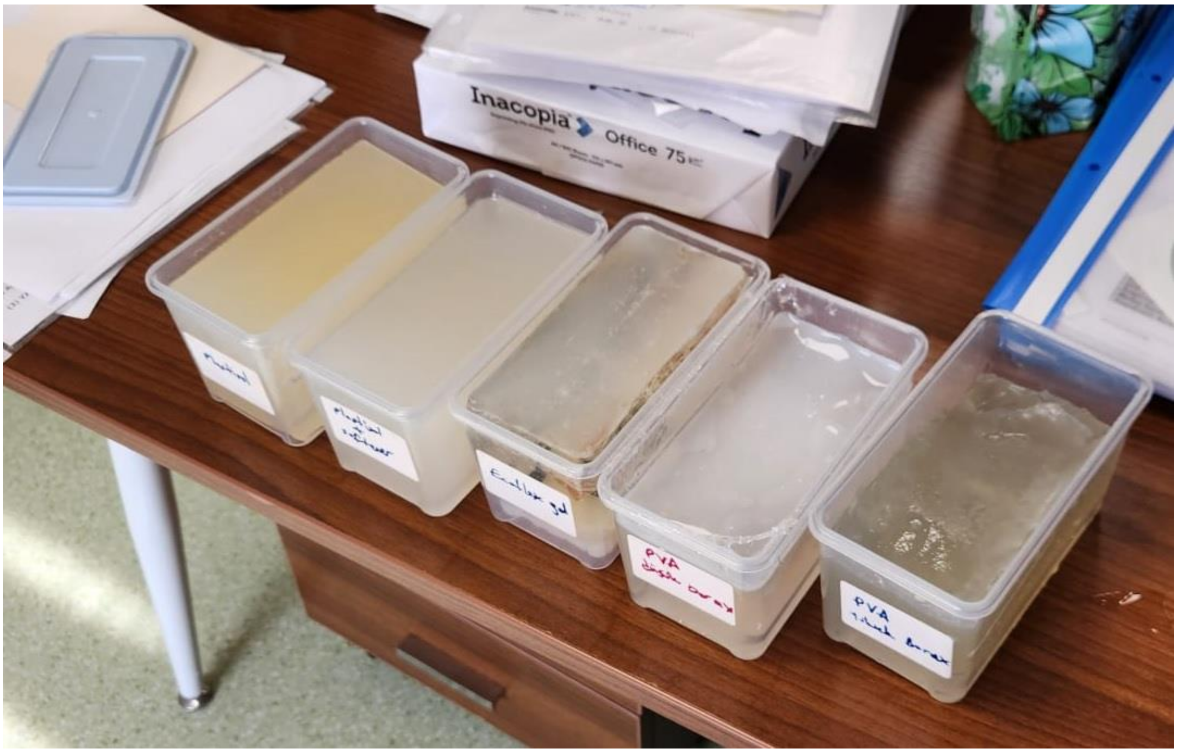

These images demonstrate the current physical condition and ultrasound imaging characteristics of all phantom materials more than one year after preparation. The photographs illustrate the macroscopic structural integrity of each material following long-term storage in sealed containers under room conditions. Corresponding ultrasound images show that the general imaging appearance, including needle visibility and background echotexture, remained comparable to initial observations.

No obvious deterioration in structural stability or imaging performance was visually identified during this follow-up assessment. These findings provide practical evidence of sustained usability over time under routine storage conditions. However, this evaluation represents an observational reassessment rather than a standardized durability testing protocol.
